# I Doubt It Is Safe: A Meta-analysis of Self-reported Intolerance of Uncertainty and Threat Extinction Training

**DOI:** 10.1016/j.bpsgos.2021.05.011

**Published:** 2021-06-05

**Authors:** Jayne Morriss, Shannon Wake, Charlotte Elizabeth, Carien M. van Reekum

**Affiliations:** Centre for Integrative Neuroscience and Neurodynamics, School of Psychology and Clinical Language Sciences, University of Reading, Reading, United Kingdom

**Keywords:** Anxiety, Exposure therapy, Extinction, Intolerance of uncertainty, Skin conductance, Threat

## Abstract

**Background:**

Intolerance of uncertainty (IU), the tendency to find uncertainty distressing, is an important transdiagnostic dimension in mental health disorders. Higher self-reported IU has been linked to poorer threat extinction training (i.e., the updating of threat to safe associations), a key process that is targeted in exposure-based therapies. However, it remains to be seen whether IU-related effects during threat extinction training are reliably and specifically driven by the IU construct or a particular subcomponent of the IU construct over other self-reported measures of anxiety.

**Methods:**

A meta-analysis of studies from different laboratories (18 experiments; sample *N* = 1006) was conducted on associations between different variants of self-reported IU (i.e., 27-item, 12-item, inhibitory, and prospective subscales), trait anxiety, and threat extinction training via skin conductance response. The specificity of IU and threat extinction training was assessed against measures of trait anxiety.

**Results:**

All the self-reported variants of IU, but not trait anxiety, were associated with threat extinction training via skin conductance response (i.e., continued responding to the old threat cue). Specificity was observed for the majority of self-reported variants of IU over trait anxiety.

**Conclusions:**

The findings suggest that the IU construct broadly accounts for difficulties in threat extinction training and is specific over other measures of self-reported anxiety. These findings demonstrate the robustness and specificity of IU-related effects during threat extinction training and highlight potential opportunities for translational work to target uncertainty in therapies that rely on threat extinction principles such as exposure therapy.

SEE COMMENTARY ON PAGE 166

The formation and adjustment of threat and safety associations are crucial for well-being and protection against psychopathology (1, 2, 3). Principles of associative threat and safety learning have provided a theoretical framework for animal and human models of the development, treatment, and relapse of anxiety, obsessive-compulsive, trauma, and stress disorders (4, 5, 6, 7, 8). Importantly, principles of associative threat and safety learning underscore modern therapies such as exposure therapy (9). Exposure-based therapies aim to reduce anxiety symptoms by gradually exposing patients to the particular objects or situations that make them feel anxious (10). The gradual exposure is thought to challenge old threat associations (i.e., “once my employer processed my paycheck and the payment was late”) by providing alternative new safe associations (i.e., “last week my employer processed my paycheck and the payment was on time”) (11). However, after exposure therapy completion, many patients experience a return of anxiety symptoms (9,12). The reason for high relapse rates after exposure therapy remains unclear. One potential factor that may hinder progress during exposure therapy is uncertainty (i.e., “when my paycheck is next processed, how can I be sure that I will be paid on time?”). Changes to contingency, such as threat to safety, may not be evident in the first instance; it may take several experiences to realize that a cue that once signaled threat now signals safety. Uncertainty (also referred to as ambiguity) over the change in contingency from threat to safety may prolong the learning of new safety associations generally (13,14), but particularly for individuals who find uncertainty anxiety provoking (15).

Intolerance of uncertainty (IU) has been defined as “an individual’s dispositional incapacity to endure the aversive response triggered by the perceived absence of salient, key, or sufficient information, and sustained by the associated perception of uncertainty” [(16), p31]. IU is a lower-order factor that underlies higher-order constructs related to negative affectivity, such as neuroticism (16,17). For example, self-reported IU accounts for unique variance in anxiety and depression symptoms when controlling for neuroticism (18,19) and has been shown to mediate the relationship between anxiety and depression symptoms and neuroticism (20). Notably, IU is a transdiagnostic dimension, with high levels of self-reported IU observed in a number of mental health disorders, such as anxiety, obsessive-compulsive, trauma, and stress disorders (20,21). Given the potential promise of IU as a transdiagnostic target for mental health interventions (22, 23, 24, 25), understanding the neurobiological basis of IU has become paramount (26,27).

From animal and human evidence, it is well established that uncertainty plays a fundamental role in the neurobiology of anxiety and stress (28, 29, 30, 31, 32, 33). However, only recently has research began to emerge on the importance of individual differences in IU in associative threat and safety learning mechanisms (27,34). Several studies, albeit from the same laboratory (34), have shown that during threat extinction training, individuals with high IU exhibit greater skin conductance response (SCR) to cues that no longer signal threat (15,35, 36, 37, 38, 39). However, presentations of disrupted threat extinction training in individuals with high IU appear to be varied. The majority of studies have found specificity of IU over other self-reported anxiety measures in predicting 1) differences in SCR to both learned threat and safety during early trials, 2) greater SCR to learned threat cues versus safety cues during late trials (36,37), or 3) greater SCR to learned threat cues across all trials (15,35). Several studies have also reported no association between IU and SCR during threat extinction training (39, 40, 41).

There is also debate concerning what self-reported IU scale or subscale is more suitable for examining cognitive, affective, and behavioral facets of anxiety (42,43). Historically, the Intolerance of Uncertainty Scale (IUS) with 27 items (IU-27) was developed to distinguish anxiety-related features in participants presenting with generalized anxiety disorder (44,45). Currently, the most prominent IUS questionnaire is the 12-item IUS (IU-12) that can be derived from the 27-item IUS (46). Both the IU-27 and the IU-12 have robust psychometrics, including good internal reliability, convergent validity, and discriminant validity (18,43,47). The IU-12 by Carleton *et al.* (46) is viewed as superior to the IU-27 because it removes high inter-item correlations and factor instability, and it divides the unilateral scale into two subscales: Prospective IU (P-IU), which refers to the desire for predictability and active seeking of certainty, and Inhibitory IU (I-IU), which refers to paralysis of cognition and action in the face of uncertainty (48). The two IUS subscales also show evidence of good internal reliability, convergent validity, and discriminant validity (18,47). An additional benefit of the two-factor scale is that it may reveal further specificity between aspects of IU and cognitive, affective, and behavioral facets of anxiety. For instance, the P-IU subscale has been specifically linked to excessive avoidance of cues that no longer signal threat (49,50). The majority of studies examining IU and threat extinction training have used the IU-27 scale (15,35, 36, 37, 38, 39,51). To further understand the role of IU in threat extinction training, however, it would be beneficial to compare associations between different scales and subscales of the IUS and indices of threat extinction training. It is possible that IU may broadly account for differences in threat extinction training or that a particular component of IU (i.e., prospective or inhibitory) is related to differences in threat extinction training.

Assessing the robustness and specificity of IU-related effects during threat extinction training could help characterize neurobiological models of uncertainty-based maintenance of anxiety (26,27), with implications for future work aiming to test, develop, or modify existing exposure-based treatments (25,52) that are more appropriate for a particular individual or group (53, 54, 55, 56). In the current study, we conducted a meta-analysis of 18 threat extinction training experiments from different laboratories (sample *N* = 1006) to examine whether IU reliably predicts threat extinction training captured via SCR [i.e., the most dominant measure used to assess threat conditioning in the literature (57)].

We compared associations between the IU-27, IU-12, and P-IU and I-IU subscales with SCR during threat extinction training (i.e., difference scores between learned threat and safety cues). Additionally, we examined associations between measures of trait anxiety [i.e., State-Trait Anxiety Inventory–Trait (STAI-T) (58) and State-Trait Inventory for Cognitive and Somatic Anxiety (STICSA) (59)] with SCR during threat extinction training. We then compared the specificity of relationships between IU and threat extinction training against measures of trait anxiety. There were several reasons for comparing IU against measures of trait anxiety. First, trait anxiety captured by STAI-T is one of the most commonly used self-reported anxiety measures in the threat conditioning literature (34). Second, the construct of trait anxiety is considered to be closely related to (60), or synonymous with, the construct of neuroticism (i.e., broader negative affect) (61), whereas IU is considered to be a lower-order factor and related to a particular part of the neuroticism construct (16) [i.e., the need for predictability or controllability (61)].

We hypothesized that the IU-27 and the IU-12 scales would be reliably and specifically associated with threat extinction training. However, given the lack of research on the IU subscales and threat extinction training, we did not have any specific hypotheses as to how the IU subscales would relate to SCR during threat extinction training.

## Methods and Materials

For the meta-analysis, PRISMA (Preferred Reporting Items for Systematic Review and Meta-Analysis) guidelines were followed (62). The protocol for the meta-analysis was not preregistered. The relevant files from the meta-analysis (i.e., search records, master data file, and meta-analysis output from R [R Foundation for Statistical Computing]) are located on the Open Science Framework through the following link: https://osf.io/8ad2q/. All aspects related to the literature search, data collation, data reduction, and data analysis were conducted independently by at least two investigators (JM, SW, and CE).

### Data Search and Inclusion Criteria

An overview of our data search is provided in the Supplement (see flowchart). First, a literature search was conducted in 4 digital databases (PubMed, bioRxiv, PsyArXiv, and Open Science Framework) using the following terms: “intolerance of uncertainty” AND (“conditioning” or “extinction”). Second, to ensure that any published or unpublished studies were not missed, datasets were called for via social media posts (i.e., Twitter) and by emailing threat conditioning experts (51 experts were contacted). The literature search and call for data were conducted between September 6, 2020, and October 16, 2020. After removing duplicate results, records were screened against the following eligibility criteria: 1) had to use a standard differential threat extinction training protocol with a conditioned stimulus (CS+) and a control stimulus (CS−), 2) had to measure SCR, and 3) had to measure self-reported IU. Next, the authors of eligible published studies were contacted regarding their willingness to share details on their design and their individual-level data (i.e., SCR, self-reported IU scores, and any additional self-reported trait anxiety scores). Based on the literature search, call for data on social media, and emailing of experts in the field, 16 records (15,35, 36, 37,39,40,51,63, 64, 65, 66, 67, 68, 69) (S. Steinman Ph.D., *et al.*, unpublished data, September 2020; R. Sjouwerman, Ph.D., and T.B. Lonsdorf, Ph.D., unpublished data, September 2020) met the inclusion criteria and were available for the meta-analysis (14 published and 2 unpublished records; 18 experiments; sample *N* = 1006) (Tables S1 and S2).

### Data Quality Check

Authors with eligible records were contacted via email and were asked to provide details of their study by completing a template spreadsheet (i.e., author list, title, sample size and characteristics, exclusion criteria, measures collected, reinforcement rate, CS type, unconditioned stimulus type, CS length, intertrial interval length, number of trials in extinction, SCR scoring type (from trough to peak; from baseline to peak), SCR value, SCR value criterion, SCR transformations, SCR nonresponse). The data quality was checked by examining whether each eligible record used an SCR extraction technique (70,71) that matched the typical recommendations for the field.

There are a variety of ways to measure and extract SCR. However, similar results for SCR have been observed when using different design choices (i.e., different interstimulus timing) (72) or preprocessing pipelines (e.g., scoring windows) (73). While there is substantial heterogeneity in the exclusion criteria used for SCR nonresponse and nonlearning in the field (74), for the current meta-analysis, SCR exclusion criteria were relatively similar across studies. The majority of records excluded participants who displayed nonresponse in SCR (i.e., none [*n* = 5], 10% [*n* = 4], or 33.3% [*n* = 3] of responses meeting SCR criterion or used no nonresponse criteria to exclude [*n* = 4]), and only 2 of 16 records excluded participants based on nonlearning in SCR. All studies were maintained in the meta-analysis.

### Data Collation

Authors of the original studies were asked to provide individual-level data for SCR, IU questionnaire (IU-27, IU-12, P-IU, or I-IU), and trait anxiety questionnaire measures in a spreadsheet with wide format. Authors were able to share trialwise or averaged SCR data and itemized or total score questionnaire data.

### Data Reduction

The data were prepared for a meta-analysis of individual participant data using a 2-stage approach (75).

#### Skin Conductance Response

The following averages were computed across SCR trialwise data: CS+ early (first 6–10 CS+ trials), CS+ late (last 6–10 CS+ trials), CS− early (first 6–10 CS− trials), and CS− late (last 6–10 CS− trials). In keeping with a variety of metrics used in the literature to capture the process of threat extinction (57), 4 separate SCR difference score metrics were computed for each experiment: whole phase extinction [(CS+) − (CS−)], early extinction [(first 6–10 CS+ trials) − (first 6–10 CS− trials)], late extinction [(last 6–10 CS+ trials) − (last 6–10 CS− trials)], and double-difference extinction score [(CS+ − CS−)_early_ − (CS+ − CS−)_late_]. While there is some interdependence between the different SCR difference scores, organizing the SCR data in this manner allows for assessment of threat and safety discrimination overall and across time (57). For 4 experiments, only the early extinction training metric was analyzed (64,65, 68) (R. Sjouwerman, Ph.D., and T.B. Lonsdorf, Ph.D., unpublished data, September 2020). This was because these studies had too few extinction learning trials to examine SCR across the whole phase, during late extinction, and comparing early versus late extinction (i.e., the studies had only 9 or 10 total trials per CS type).

#### Intolerance of Uncertainty

Scores from 4 separate scales (IU-27, IU-12, I-IU, and P-IU) were generated from the IUS (Table S3) (44). In the original IUS, the 27 items are rated on a 5-point Likert scale. The IU-12 score is generated from 12 items from the IUS. Two experiments administered only the IU-12 (40,63) and therefore are not included in the analysis of the IU-27. The I-IU and P-IU are two subscales measuring separate components of IU and are generated from either the IU-27 or the IU-12. Where 2 or more items were missing for the IUS, values were interpolated based on the average item score (*n* = 14).

#### Trait Anxiety

Of the 18 studies, 15 measured trait anxiety using the STAI-T or the STICSA as an alternative self-report measure of anxiety. The STAI-T (58) consists of 20 trait anxiety items rated on a 4-point Likert scale. The STICSA (59) consists of 21 items that are rated on a 4-point Likert scale.

### Analyses

Correlation and partial correlation analyses were performed in SPSS 19 (IBM Corp.) for each dataset. To examine whether IU or trait anxiety was related to threat extinction training in each experiment, correlations were conducted between the variants of the IUS (IU-27, IU-12, I-IU, and P-IU), variants of trait anxiety (STAI-T or STICSA), and SCR difference scores (whole phase, early, late, and double-difference) during the threat extinction training phase (Tables S4 and S5). To assess the specificity of IU over other self-report measures of anxiety in each experiment, partial correlations were conducted between the IUS variants (IU-27, IU-12, I-IU, and P-IU) and SCR difference scores (whole phase, early, late, and double-difference) during the threat extinction training phase, controlling for STAI-T or STICSA (Table S6).

The *r* values from the correlations and partial correlations were converted into Hedges’ *g* effect size values. Fixed-effect meta-analyses were carried out in RStudio (RStudio, Inc., Boston, MA) on the effect sizes from the correlations and partial correlations separately to generate a pooled effect size for every IU scale/subscale (IU-27, IU-12, I-IU and, P-IU), trait anxiety variant (STAI-T, STICSA), and difference score (early, late, whole phase, and double-difference) across the 18 experiments. Benjamini-Hochberg corrections (76) were applied to the correlations (corrected value, *p* < .025) and partial correlations (corrected value, *p* < .018).

## Results

### Relationships Between IU and SCR During Threat Extinction

**Figure 1:**
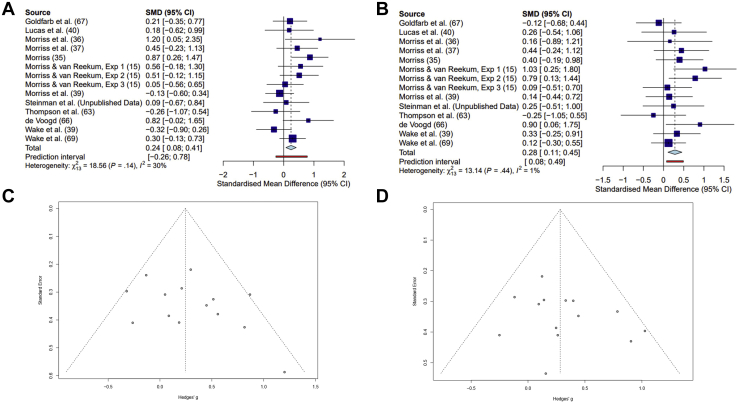
Forest plots demonstrating a small-to-medium effect size across studies for the relationships between the 12-item Intolerance of Uncertainty Scale and skin conductance response difference scores (CS+ − CS−) during late extinction **(A)** and across the entire extinction phase **(B)**. Funnel plots were symmetrical, indicating little publication bias for studies in the meta-analyses examining the relationship between 12-item Intolerance of Uncertainty Scale and skin conductance response difference scores during late extinction **(C)** and across the entire extinction phase **(D)**. In sum, individuals with higher intolerance of uncertainty continue to respond to learned threat cues in the absence of reinforced threat, indicating difficulty in updating threat associations to safe associations. Note that 4 experiments were not included owing to a lack of trials for late and entire extinction phase analysis. CI, confidence interval; CS, conditioned stimulus; SMD, standardized mean difference.

All the self-reported variants of the IUS (IU-27, IU-12, I-IU, and P-IU) were significantly associated with SCR difference scores during late extinction training and across the entire extinction phase (corrected *p*s < .025) (Table 1). The significant meta-analytic effect sizes for relationships between self-reported IU and SCR differences scores during late extinction training and the entire extinction phase were small to medium (Hedges’ *g* 0.2–0.35) (Table 1 and Figure 1A, B) and yielded fairly low heterogeneity across studies (*I*^2^ 0–32.5%) (Table 1).

**Table 1:** Pooled Effect Sizes and Heterogeneity^a^ for the Scales and Difference Scores for SCR During Extinction

	*g*	95% CI	k	*n*	*p*	*I*^2^
IU-27 Scale						
Early ext	−0.01	−0.14, 0.11	16	958	.822	45.6%
Late ext	0.35	0.17, 0.53	12	504	<.001	17.2%
Whole phase ext	0.29	0.11, 0.46	12	504	.001	1.5%
Double-difference	−0.28	−0.45, −0.1	12	504	.002	31.8%
IU-12 Scale						
Early ext	0.06	−0.06, 0.19	18	1006	.341	44.9%
Late ext	0.24	0.08, 0.41	14	552	.005	29.9%
Whole phase ext	0.28	0.11, 0.45	14	552	.001	1.1%
Double-difference	−0.12	−0.28, 0.05	14	552	.180	38.2%
I-IU Scale						
Early ext	0.03	−0.09, 0.16	18	1006	.621	40.4%
Late ext	0.25	0.08, 0.42	14	552	.004	10.9%
Whole phase ext	0.22	0.05, 0.39	14	552	.010	0%
Double-difference	−0.15	−0.32, 0.02	14	552	.090	43%
P-IU Scale						
Early ext	0.07	−0.06, 0.19	18	1006	.301	42%
Late ext	0.20	0.04, 0.37	14	552	.017	32.5%
Whole phase ext	0.23	0.06, 0.39	14	552	.008	10%
Double-difference	−0.08	−0.25, 0.09	14	552	.348	20%

CI, confidence interval; ext, extinction; I-IU, Inhibitory IU; IU, intolerance of uncertainty; P-IU, Prospective IU; SCR, skin conductance response.

a Percentage of variability in effect size.

Only the IU-27 (not IU-12, I-IU, or P-IU) was significantly associated with SCR double-difference scores during extinction training (corrected *p* < .025). None of the self-reported variants of the IUS (IU-27, IU-12, I-IU, and P-IU) were significantly associated with SCR difference scores during early extinction training (*p*s > .3) (Table 1 and Table S7).

### Relationships Between Trait Anxiety and SCR During Threat Extinction

No significant relationships were found between trait anxiety and SCR difference scores (corrected *p* > .1) (Table 2).

**Table 2:** Pooled Effect Sizes and Heterogeneity^a^ for the Trait Anxiety Scores and Difference Scores for SCR During Extinction

	*g*	95% CI	k	*n*	*p*	*I*^2^
Trait Anxiety						
Early ext	0.016	−0.11, 0.15	15	920	.805	8.5%
Late ext	0.043	−0.14, 0.22	11	479	.638	0%
Whole phase ext	0.146	−0.03, 0.325	11	479	.112	0%
Double-difference	−0.017	−0.2, 0.16	11	479	.852	21.1.%

CI, confidence interval; ext, extinction; SCR, skin conductance response.

a Percentage of variability in effect size.

### Relationships Between IU and SCR During Threat Extinction When Controlling for Measures of Trait Anxiety

Even though trait anxiety was not significantly related to threat extinction training, it is important to establish specificity of IU over trait anxiety owing to shared variance between these constructs (correlation between IU-12 and STAI-T, *r*_802_ = 0.568, *p* < .001; correlation between IU-12 and STICSA, *r*_115_ = 0.217, *p* = .003). When controlling for trait anxiety, IU-12, P-IU, and I-IU (but not IU-27) were significantly associated with SCR difference scores during late extinction training (corrected *p* < .018) (Table 3). Moreover, when controlling for trait anxiety, IU-27, IU-12, and P-IU (but not I-IU) were significantly associated with SCR difference scores across the entire extinction phase (corrected *p* < .018) (Table 3). The meta-analytic effect sizes for significant relationships between self-reported IU and SCR differences scores during late extinction training and the entire extinction phase, when controlling for self-reported trait anxiety, were small to medium (Hedges’ *g* 0.24–0.31) (Table 3) and showed fairly low heterogeneity across studies (*I*^2^ 0–26.9%) (Table 3). When controlling for trait anxiety, none of the self-reported variants of the IUS (IU-27, IU-12, I-IU, and P-IU) were significantly associated with SCR double-difference scores (corrected *p*s between .029 and .06) or SCR difference scores during early extinction training (corrected *p*s > .3) (Table 3).

**Table 3:** Pooled Effect Sizes and Heterogeneity^a^ for IU Scales and Difference Scores for SCR During Extinction When Controlling for Trait Anxiety Scores^b^

	*g*	95% CI	k	*n*	*p*	*I*^2^
IU-27 Scale						
Early ext	−0.03	−0.16, 0.1	15	933	.661	45.3%
Late ext	0.14	−0.04, 0.33	11	479	.120	65.5%
Whole phase ext	0.31	0.13, 0.49	11	479	<.001	18.8%
Double-difference	−0.21	−0.39, −0.02	11	479	.029	52.1%
IU-12 Scale						
Early ext	0.04	−0.08, 0.17	15	933	.498	46.1%
Late ext	0.28	0.10, 0.47	11	479	.002	26.9%
Whole phase ext	0.25	0.07, 0.43	11	479	.007	14%
Double-difference	−0.17	−0.35, 0.01	11	479	.066	43%
I-IU Scale						
Early ext	0.004	−0.12, 0.13	15	933	.947	41.2%
Late ext	0.29	0.11, 0.47	11	479	.002	18%
Whole phase ext	0.18	0.002, 0.36	11	479	.047	16.1%
Double-difference	−0.18	−0.37, −0.003	11	479	.046	51.6%
P-IU Scale						
Early ext	0.06	−0.07, 0.19	15	933	.354	32.8%
Late ext	0.23	0.05, 0.41	11	479	.013	22%
Whole phase ext	0.24	0.06, 0.42	11	479	.009	0%
Double-difference	−0.13	−0.31, 0.05	11	479	.163	19.3%

CI, confidence interval; ext, extinction; I-IU, Inhibitory IU; IU, intolerance of uncertainty; P-IU, Prospective IU; SCR, skin conductance response.

a Percentage of variability in effect size.

b State-Trait Anxiety Inventory–Trait or State-Trait Inventory for Cognitive and Somatic Anxiety.

### Moderator Analyses

Moderator analyses with laboratory group (University of Reading vs. other institutions) as a factor were conducted on the effect sizes of the relationship between IU-12 and SCR difference scores during late extinction training and the entire extinction training phase. The laboratory group factor did not significantly moderate the effect sizes of the relationship between IU-12 and SCR difference scores during late extinction training (*Q*_1_ = 0.23, *p* = .63) (Table 4) or the entire extinction training phase (*Q*_1_ = 0.68, *p* = .41) (Table 4).

**Table 4:** Moderator Analysis Demonstrating Pooled Effect Sizes and Heterogeneity^a^ Across Subgroup for IU-12 and SCR During Late Extinction Training and Entire Extinction Training Phase

	Subgroup	*g*	95% CI	k	*I*^2^	*Q*	*p*
Late ext	University of Reading	0.303	0.02, 0.58	9	46.60%	0.23	.63
	Other	0.197	−0.13, 0.52	5	0.00%		
Whole phase ext	University of Reading	0.337	0.13, 0.54	9	0.00%	0.68	.41
	Other	0.159	−0.21, 0.53	5	20.70%		

CI, confidence interval; ext, extinction; IU-12, 12-item Intolerance of Uncertainty Scale; SCR, skin conductance response.

a Percentage of variability in effect size.

### Publication Bias Assessment

Publication bias was assessed using Egger’s regression tests and funnel plots for the two most prominent results across the meta-analyses conducted, i.e., meta-analyses examining the relationship between IU-12 and SCR difference scores during late extinction training and the entire extinction training phase. Egger’s tests were not significant for the studies included in meta-analyses examining the relationship between IU-12 and SCR difference scores during late extinction training (*t* = 1.42, *p* = .18) or across the entire extinction training phase (*t* = 1.19, *p* = .26). This result and the symmetry of the funnel plots presented in Figure 1C and D suggest that there is very little evidence for publication bias.

## Discussion

Threat extinction is a key principle underlying exposure-based therapies (5). In this study, in a meta-analysis of 18 experiments, we show that IU, the tendency to find uncertainty distressing (16,17,44), consistently and specifically impairs threat extinction training, indexed by greater SCR to cues that no longer signal threat. The findings consolidate the role of IU-related biases in threat extinction training and have clear implications for neurobiological models of uncertainty-related maintenance of anxiety (27,31,32) and future translational work aiming to target IU in exposure-based therapies (26).

All the self-reported variants of the IUS were associated with greater SCR to learned threat versus safety cues 1) across threat extinction training and 2) during the late trials of threat extinction training. No relationships were found between trait anxiety (STAI and STICSA) and SCR difference scores metrics during threat extinction training. Importantly, the majority of the IU-related effects during threat extinction training remained, particularly for the IU-12, when controlling for self-reported measures of trait anxiety such as STAI and STICSA. The meta-analysis suggests that the IU construct broadly, and not a subcomponent of the IU construct (i.e., prospective or inhibitory), accounts for difficulties in updating threat to safety. Such findings support prior work suggesting that the IUS is best represented by the total score (IUS-12 item), rather than the subscales (i.e., P-IU or I-IU) (42,77). Furthermore, the meta-analysis revealed that IU is associated with updating threat to safety, over other self-report measures of trait anxiety (i.e., STAI and STICSA). These findings suggest that the IU construct, which is in part related to the need for predictability/controllability facet of the neuroticism construct (16), is more predictive of threat extinction learning than trait anxiety constructs that strongly overlap with multiple facets of the neuroticism construct (60,61).

In previous research, a few studies observed differences in SCR to both learned threat and safety cues during the early trials of threat extinction training (36,37). However, the meta-analysis showed that none of the self-reported variants of IU were reliably associated with SCR to learned threat or safety cues during the early trials of threat extinction training. During the early part of extinction training, participants begin to learn that the CS+ is no longer being reinforced with an aversive outcome (i.e., a shock or loud sound). However, the time it takes participants to learn that a contingency change has occurred may differ depending on the reinforcement rate used during the prior acquisition phase (78,79). Notably, the original studies that found a relationship between IU and early extinction used a 100% reinforcement rate during acquisition (36,37), where the change in contingencies between acquisition and extinction are more obvious. Within the current meta-analysis, the reinforcement rates during acquisition varied substantially across studies (i.e., 37.5%–100%), which may explain the lack of IU-related effects during early extinction.

While the IU-related effects across extinction training by time may seem unintuitive, given that contingency uncertainty may be greatest during early extinction training, the findings are in line with modern IU theory. Based on Carleton’s definition of IU (16), aversive responses triggered by the perceived absence of information are sustained by the perception of uncertainty. In the case of extinction learning, individuals with high IU relative to low IU may sustain the perception of uncertainty for longer, resulting in the maintenance of a conditioned response. Indeed, the lack of information about the omission of threat throughout the extinction phase may cumulatively add to the perception of uncertainty in individuals with high IU (i.e., “I didn’t hear the sound on the last trial. Maybe it will happen on the next trial?”).

Importantly, the findings from the present meta-analysis highlight the relevance of IU in threat extinction training, a key process that is targeted in exposure-based therapies (5, 6, 7), and therefore has implications for clinical work. A next step for experimental work would be to identify the extent to which IU-related difficulties in threat extinction training are transdiagnostic using a Research Domain Criteria approach (53,55,56). For instance, it is unclear whether IU is associated with poorer threat extinction training within a specific or broader cluster of mental health disorders. Translational research could identify whether existing evidence-based therapies such as cognitive behavioral therapy (i.e., which often involve exposure sessions with cognitive restructuring techniques) are effective in reducing IU-related biases across disorders or whether further modification (i.e., prolonged exposure-based therapy) to these treatments (25,52) are needed to target IU-related biases in a particular disorder or within a broader set of disorders (26). Answering these questions would allow for precision psychiatry (54), where clinicians could select a particular type of therapy for individuals with high IU in disorders or cases in which it is relevant.

The meta-analysis had several strengths. First, the meta-analysis data were more heterogeneous than typically found from a single study (i.e., data from different laboratories and from different sample types). Second, despite differences in design and data reduction techniques, IU was still the dominant anxiety construct in predicting threat extinction training behavior via SCR, suggesting that IU-related effects are particularly robust in the face of additional noise and error variance. Third, the results of the meta-analysis were not moderated by laboratory group, suggesting that IU-related effects during threat extinction training via SCR are not limited to a particular set of researchers, laboratory setup, or sample demographic.

Future work should focus efforts on replicating IU-related effects in non–English speaking countries, in non–Western, educated, industrialized, rich, and democratic samples (80), and in other readout measures (57) to further assess the generalizability, reliability, and specificity of IU-related effects during threat extinction training. Furthermore, the meta-analyses primarily used data from same-day or next-day uninstructed threat extinction training with only one session, limiting the generalizability of the results to real-world exposure-based therapies (i.e., which typically involve instructions about assessing the likelihood of aversive events and often comprise more than one session) (8). Promisingly, several studies have shown that higher IU is associated with poorer threat extinction retention 24 hours later and that this can be alleviated either by extending the session (i.e., longer session) or by introducing a novel stimulus (e.g., pairing the CS+ with a benign tone) during threat extinction training (41,51). However, further research is needed to assess the impact and stability of IU-related effects across more extinction training sessions and extended periods of time (51) as well as to identify whether biological mechanisms modulated by IU can be altered via therapeutic and/or pharmacological interventions to extrapolate the clinical relevance of IU in the treatment of anxiety and stress disorders (26).

Overall, the findings from the meta-analysis demonstrate the robustness and specificity of IU-related effects during threat extinction training. Furthermore, the findings highlight potential opportunities for experimental and translational work to examine how IU modulates threat extinction learning across different disorders with an anxiety component and whether existing therapies that rely on threat extinction principles (i.e., exposure therapy) need to be modified to target IU-related biases.
