# Supplementary material for: I Doubt It Is Safe: A Meta-analysis of Self-reported Intolerance of Uncertainty and Threat Extinction Training
Source: Biol Psychiatry Glob Open Sci. 2021 Jun 5;1(3):171–9. doi: 10.1016/j.bpsgos.2021.05.011 (PMC9616306; doi:10.1016/j.bpsgos.2021.05.011)
Supplement: Supplementary Materials [file mmc1.pdf]

I Doubt It Is Safe: A Meta-analysis of Self-reported Intolerance of Uncertainty and Threat Extinction Training

Supplemental Information

Supplementary Table S1.

Study characteristics and experimental parameters

Study	IU Scale Administered	Trait Anxiety Measure Administered	Reinforcement Rate	Instruction Type	CS Type	US Type	CS Length (ms)	ITI Length (ms)	N Trials Extinction	SCR Scoring Window (ms after trial onset)
Goldfarb et al. (67)	IU-27	STAI-T	73%	Uninstructed	Tones and coloured squares	Electric shock	6000	8000 – 10000	24 (12+, 12 CS-)	500 - 6000
Kanen et al. (68)	IU-27	STAI-T	37.5%	Uninstructed	Coloured squares	Electric shock	4000	10000	20 (10 CS+, 10 CS-)	500 - 4500
Lucas et al. (40)	IU-12	N/A	50%	Uninstructed	Angry male caucasian faces	Electric shock	8000	22000, 24000 or 26000	32 (16 CS+, 16 CS-)	1000 – 4000
Morriss et al. (36)	IU-27	STAI-T	100%	Uninstructed	Coloured squares	Female scream	1500	3000 – 6450	32 (16 CS+, 16 CS-)	0 – 7000
Morriss et al. (37)	IU-27	STAI-T	100%	Uninstructed	Coloured squares	Female scream	1500	3000 – 6450	32 (16 CS+, 16 CS-)	0 – 7000
Morriss & van Reekum, Exp 1 (15)	IU-27	STAI-T	50%	Uninstructed	Coloured squares	Female scream	4000	6000 – 8800	32 (16 CS+, 16 CS-)	500 – 3500

Morriss & van Reekum, Exp 2 (15)	IU-27	STAI-T	50%	Uninstructed	Coloured squares	Female scream	4000	6000 – 8800	32 (16 CS+, 16 CS-)	500 – 3500
Morriss & van Reekum, Exp 3 (15)	IU-27	STAI-T	50%	Uninstructed	Coloured squares	Female scream	4000	6000 – 8800	32 (16 CS+, 16 CS-)	500 – 3500
Morriss (35)	IU-27	STAI-T	50%	Uninstructed	Coloured squares	Female scream	4000	6000 – 8800	32 (16 CS+, 16 CS-)	500 – 3500
Morriss et al. (38)	IU-27	STICSA	50%	Uninstructed	Coloured squares	Female scream	4000	6000 – 8800	32 (16 CS+, 16 CS-)	500 – 3500
Sjouwerman et al. (64)	IU-27	STAI-T	100%	Uninstructed	Black shapes (i.e. grid or spiral) on a background picture of water	Electric shock	6000	10000 – 13000	18 (9 CS+, CS-)	900 - 4000
Sjouwerman et al. (65)	IU-27	STAI-T	100%	Uninstructed	Black geometrical symbols on coloured background	Electric shock	6000	10000 - 13000	18 (9 CS+, CS-)	900 - 4000
Sjouwerman & Lonsdorf (Unpublished Data)	IU-27	STAI-T	100%	Uninstructed	Black geometrical symbols on coloured background	Electric shock	6000	11000 - 13000	18 (9 CS+, CS-)	900 - 3500
Steinman et al. (Unpublished Data)	IU-27	STAI-T	53.33%	Uninstructed	Angry male caucasian faces	Electric shock	6000	12000	40 (20 CS+, 20 CS-)	500 - 6000
Thompson et al. (63)	IU-12	N/A	100%	Uninstructed	Coloured images of animals (fish and birds)	Electric shock	6000	13000 - 17000	24 (12 CS+, 12 CS-)	1000 - 6000

de Voogd et al. (66)	IU-27	N/A	37.50%	Uninstructed	Pictures of snakes	Electric shock	6000	18000 – 22000	40 (20 CS+, 20 CS-)	500 - 6500
Wake et al. (39)	IU-27	STICSA	50%	Uninstructed	Coloured squares	Female scream	4000	6000 – 8800	32 (16 CS+, 16 CS-)	500 – 3500
Wake et al. (69)	IU-27	STAI-T	50%	Uninstructed	Neutral female caucasian faces	Electric shock and critical verbal statements	4000	6000 – 8800	32 (16 CS+, 16 CS-)	500 – 3500

STAI-T, State-Trait Anxiety Inventory-Trait; STICSA, The State-Trait Inventory for Cognitive and Somatic Anxiety; CS, Conditioned stimulus; US, Unconditioned stimulus; N, Number; ITI, Inter-trial interval.

Supplementary Table S2

Sample characteristics across studies

Study	Sample Type	Sex	Ethnicity	Age
Goldfarb et al. (67)	Community and students	30 F / 18 M	Not recorded	22.25
Kanen et al. (68)	Healthy	18 F / 29 M	Data not returned on time	25
Lucas et al. (40)	Community and students	29 F / 19 M (across exp groups)	15 Caucasian, 7 Asian, 2 Indian	25 (across exp groups)
Morriss et al. (36)	Community and students	12 F / 10 M	18 White, 2 Asian, 2 Mixed	23.59
Morriss et al. (37)	Students	32 F / 6 M	Not recorded	18-25 years
Morriss & van Reekum, Exp 1 (15)	Community and students	33 F / 27 M (across exp groups)	Not recorded	23.56 (across exp groups)
Morriss & van Reekum, Exp 2 (15)	Community and students	57 F / 24 M (across exp groups)	Not recorded	24.65 (across exp groups)
Morriss & van Reekum, Exp 3 (15)	Students	86 F / 11 M (across exp groups)	72 White, 13 Asian, 6 Black, 4 Mixed, 2 Middle Eastern (across exp groups)	20.61 (across exp groups)
Morriss (35)	Community and students	31 F / 14 M	33 White, 5 Asian, 4 Black, 3 Mixed	23
Morriss et al. (38)	Community and students	86 F / 58 M (across exp groups)	92 White, 29 Asian, 15 not specified, 4 Middle Eastern/Arab, 2 Black, 2 Mixed (across exp groups)	24 (across exp groups)
Sjouwerman et al. (64)	Community and students	255 F / 101 M	Not recorded	25
Sjouwerman et al. (65)	Community and students	38 F / 19 M	Not recorded	25
Sjouwerman & Lonsdorf (Unpublished Data)	Community and students	66 F / 22 M	Not recorded	25

Steinman et al. (Unpublished Data)	Clinically diagnosed with anxiety or obsessive compulsive disorder	15 F / 12 M	17 White, 4 Other, 3 Black, 3 Asian	24.33
Thompson et al. (63)	Students	15 F / 9 M	15 Caucasian, 7 Asian, 2 Indian	20.37
de Voogd et al. (66)	Healthy, students	61 F/ 41 M (across exp groups)	35 Asian/Asian Americans, 29 Caucasian/White, 20 Black/African American, 8 Mixed, 5 Unknown/not indicated, 3 Hispanic non-white, 1 Hispanic White, 1 Arab (across exp groups)	24.4 (across exp groups)
Wake et al. (39)	Community and students	67 F / 28 M (across exp groups)	61 White, 14 not specified, 10 Asian, 3 Middle Eastern/Arab, 2 Black, 1 Mixed (across exp groups).	24.4 (across exp groups)
Wake et al. (69)	Students	84 F	67 White, 14 Asian/Pacific Islander, 7 Black, 3 Mixed, 1 Middle Eastern/Arab	19.66

Supplementary Table S3.

Summary of means (M), standard deviations (SD) and range of scores for each IU-scale in the eighteen experiments.

Study	N	IU-27			IU-12			I-IU			P-IU		
		M	SD	Range	M	SD	Range	M	SD	Range	M	SD	Range
Goldfarb et al. (67)	49	63.00	14.59	29 - 100	28.63	7.67	14-46	9.94	3.48	5-20	18.69	5.19	9-31
Kanen et al. (68)	47	52.57	13.74	31-85	24.70	7.17	13-39	8.15	2.77	5-15	16.55	5.09	8-25
Lucas et al. (40)	24	N/A	N/A	N/A	32.92	9.13	18-55	11.54	4.67	5-23	21.38	5.09	12-32
Morriss et al. (36)	14	50.86	16.32	27-85	24.14	8.28	12-39	8.36	2.71	5-13	15.79	6.36	7-26
Morriss et al. (37)	34	62.88	18.99	31-116	28.5	9.1	12-52	10.41	4.16	5-21	18.1	5.93	7-31
Morriss & van Reekum, Exp 1 (15)	29	66.66	16.83	33-94	30.41	7.95	15-46	11.14	3.32	5-17	19.28	4.95	10-31
Morriss & van Reekum, Exp 2 (15)	39	66.21	17.46	33-104	31.64	9.3	15-51	11.31	3.8	5-18	20.33	6.61	9-35
Morriss & van Reekum, Exp 3 (15)	42	63.36	17.61	33-103	29.48	9.17	15-48	10.07	3.67	5-19	19.4	6.16	10-32

Morriss (35)	46	64.15	17.36	31-94	29.96	8.22	14-45	10.63	3.33	5-18	19.33	5.56	9-29
Morriss et al. (38)	70	64.53	19.82	32-122	29.23	8.57	16-58	10.56	4.1	5-25	18.67	5.34	9-33
Sjouwerman et al. (64)	54	57.65	18.38	27-121	26.70	8.74	12-55	10.35	3.80	5-24	16.35	5.67	7-31
Sjouwerman et al. (65)	267	62.77	18.44	27-135	28.62	8.74	12-60	11.18	4.12	5-25	17.44	5.45	7-35
Sjouwerman & Lonsdorf (Unpublished Data)	86	53.49	14.74	27-93	24.50	7.02	12-45	9.06	2.93	5-18	15.44	4.74	7-27
Steinman et al. (Unpublished Data)	27	75.22	19.82	42-117	34.41	8.86	19-52	13.11	4.53	6-21	21.30	5.14	12-31
Thompson et al. (63)	24	N/A	N/A	N/A	28.00	8.39	16-47	9.54	3.56	5-18	18.46	5.12	11-29
de Voogd et al. (66)	24	58.17	19.89	32-103	27.25	9.00	17-47	9.04	3.61	5-17	18.21	5.94	10-30

Wake et al. (39)	46	64.21	21.9	28-110	29.15	10.39	12-52	10.64	3.96	5-18	18.51	6.86	7-34
Wake et al. (69)	84	64.68	17.92	35-113	29.61	8.31	15-49	10.89	3.56	5-19	18.71	5.49	8-33

Supplementary Table S4.
Correlations between the IU Subscales (IU-27, IU-12, I-IU and P-IU) and difference scores (early, late, whole phase and double difference) for SCR during extinction training

Study	Difference Score	IU 27	IU 12	I IU	P IU
Goldfarb et al. (67)	Early	-0.20	-0.18	-0.24	-0.11
	Late	0.17	0.11	0.12	0.08
	Whole Phase	-0.02	-0.06	-0.10	-0.02
	Double Difference	-0.24	-0.19	-0.23	-0.12
Kanen et al. (68)	Early	-0.04	-0.06	-0.11	-0.02
	Late	NA	NA	NA	NA
	Whole Phase	NA	NA	NA	NA
	Double Difference	NA	NA	NA	NA
Lucas et al. (40)	Early	NA	0.14	0.25	0.03
	Late	NA	0.10	0.17	0.01
	Whole Phase	NA	0.13	0.24	0.02
	Double Difference	NA	0.05	0.07	0.02
Morriss et al. (36)	Early	-0.37	-0.38	-0.39	-0.33
	Late	0.66*	0.54*	0.63*	0.44
	Whole Phase	0.19	0.08	0.14	0.05
	Double Difference	-0.65*	-0.58*	-0.64*	-0.49
Morriss et al. (37)	Early	-0.37*	-0.40*	-0.36*	-0.35*
	Late	0.17	0.22	0.26	0.16
	Whole Phase	0.27	0.22	0.28	0.14
	Double Difference	-0.36*	-0.41*	-0.41*	-0.34
Morriss (35)	Early	-0.06	-0.11	-0.24	-0.02
	Late	0.36*	0.40**	0.32*	0.40**
	Whole Phase	0.21	0.20	0.042	0.27
	Double Difference	-0.25	-0.31*	-0.36*	-0.25
	Early	0.41*	0.47*	0.40*	0.47*

Morriss & van Reekum (15)	Late	0.22	0.31	0.26	0.31
	Whole Phase	0.40*	0.48*	0.42*	0.48*
	Double Difference	0.20	0.19	0.16	0.19
Morriss & van Reekum (15)	Early	0.21	0.23	0.17	0.22
	Late	0.28	0.27	0.25	0.23
	Whole Phase	0.36*	0.36*	0.30	0.33*
	Double Difference	-0.05	-0.03	-0.06	-0.01
Morriss & van Reekum (15)	Early	0.07	0.05	0.07	0.03
	Late	-0.01	0.01	-0.07	0.07
	Whole Phase	0.03	0.03	-0.10	0.06
	Double Difference	0.06	0.03	0.11	-0.02
Morriss et al. (38)	Early	-0.19	0.20	0.18	0.19
	Late	0.13	-0.06	-0.01	-0.09
	Whole Phase	-0.04	0.09	0.10	0.06
	Double Difference	-0.25*	0.20	0.12	0.22
Sjouwerman et al. (64)	Early	-0.1	-0.11	-0.02	-0.15
	Late	NA	NA	NA	NA
	Whole Phase	NA	NA	NA	NA
	Double Difference	NA	NA	NA	NA
Sjouwerman et al. (65)	Early	0.07	0.10	0.10	0.10
	Late	NA	NA	NA	NA
	Whole Phase	NA	NA	NA	NA
	Double Difference	NA	NA	NA	NA
Sjouwerman & Lonsdorf (Unpublished Data)	Early	-0.12	-0.12	-0.16	-0.09
	Late	NA	NA	NA	NA
	Whole Phase	NA	NA	NA	NA
	Double Difference	NA	NA	NA	NA
Steinman et al. (Unpublished Data)	Early	0.32	0.18	0.13	0.19
	Late	0.20	0.04	-0.01	0.08
	Whole Phase	0.30	0.13	0.07	0.16
	Double Difference	0.10	0.12	0.13	0.09

Thompson et al. (63)	Early	NA	-0.08	-0.10	-0.07
	Late	NA	-0.14	-0.06	-0.18
	Whole Phase	NA	-0.13	-0.10	-0.14
	Double Difference	NA	0.02	-0.05	0.07
de Voogd et al. (66)	Early	0.36	0.40	0.22	0.47*
	Late	0.33	0.39	0.26	0.43*
	Whole Phase	0.37	0.42*	0.26	0.49*
	Double Difference	0.08	0.06	-0.03	0.10
Wake et al. (39)	Early	-0.003	0.08	0.06	0.09
	Late	-0.14	-0.16	-0.07	-0.19
	Whole Phase	0.11	0.17	0.09	0.20
	Double Difference	-0.12	-0.08	-0.02	-0.10
Wake et al (69)	Early	-0.06	-0.04	-0.02	-0.04
	Late	0.2	0.15	0.15	0.13
	Whole Phase	0.08	0.06	0.08	0.04
	Double Difference	-0.16	-0.11	-0.10	-0.11

** Correlation significant at the 0.01 level (2-tailed)
*Correlation significant at the 0.05 level (2-tailed)

Supplementary Table S5.
Partial correlations between the IU Subscales (IU-27, IU-12, I-IU and P-IU) and difference scores (early, late, whole phase and double difference) for SCR during extinction training when controlling for trait anxiety scores (STAI-T and STICSA)

Study	Difference Score	IU 27	IU 12	I IU	P IU
Goldfarb et al. (67)	Early	-0.22	-0.20	-0.27	-0.11
	Late	0.23	0.15	0.19	0.10
	Whole Phase	0.01	-0.03	-0.07	-0.01
	Double Difference	-0.28	-0.22	-0.29*	-0.14
Kanen et al. (68)	Early	0.08	0.03	0.001	0.04
	Late	NA	NA	NA	NA
	Whole Phase	NA	NA	NA	NA
	Double Difference	NA	NA	NA	NA
Lucas et al. (40)	Early	NA	NA	NA	NA
	Late	NA	NA	NA	NA
	Whole Phase	NA	NA	NA	NA
	Double Difference	NA	NA	NA	NA
Morriss et al. (36)	Early	-0.16	-0.23	-0.20	-0.21
	Late	0.61*	0.43	0.55	0.33
	Whole Phase	0.30	0.12	0.23	0.07
	Double Difference	-0.50	-0.44	-0.49	-0.36
Morriss et al. (37)	Early	-0.22	-0.26	-0.21	-0.21
	Late	0.08	0.17	0.23	0.08
	Whole Phase	0.36*	0.27	0.35*	0.13
	Double Difference	-0.20	-0.29	-0.30	-0.19
Morriss (35)	Early	-0.18	-0.19	-0.35*	-0.07
	Late	0.45**	0.45**	0.38*	0.44**
	Whole Phase	0.17	0.16	-0.02	0.25
	Double Difference	-0.39*	-0.40**	-0.47**	-0.31
	Early	0.39*	0.44*	0.34	0.44*

Morriss & van Reekum (15)	Late	0.24	0.34	0.27	0.33
	Whole Phase	0.40*	0.49**	0.38*	0.48**
	Double Difference	0.16	0.13	0.10	0.13
Morriss & van Reekum (15)	Early	0.32	0.28	0.22	0.25
	Late	0.31	0.27	0.25	0.22
	Whole Phase	0.46*	0.40*	0.34*	0.34*
	Double Difference	-0.01	0.004	-0.03	0.02
Morriss & van Reekum (15)	Early	-0.10	-0.11	-0.08	-0.10
	Late	0.02	0.04	-0.07	0.09
	Whole Phase	-0.06	-0.05	-0.10	-0.01
	Double Difference	-0.09	-0.11	-0.01	-0.15
Morriss et al. (38)	Early	-0.24*	0.20	0.18	0.19
	Late	0.05	-0.06	-0.01	-0.08
	Whole Phase	-0.12	0.09	0.11	0.07
	Double Difference	-0.23	0.20	0.15	0.21
Sjouwerman et al. (64)	Early	-0.19	-0.20	-0.10	-0.23
	Late	NA	NA	NA	NA
	Whole Phase	NA	NA	NA	NA
	Double Difference	NA	NA	NA	NA
Sjouwerman et al. (65)	Early	0.09	0.12*	0.11	0.11
	Late	NA	NA	NA	NA
	Whole Phase	NA	NA	NA	NA
	Double Difference	NA	NA	NA	NA
Sjouwerman & Lonsdorf (Unpublished Data)	Early	-0.07	-0.08	-0.13	-0.05
	Late	NA	NA	NA	NA
	Whole Phase	NA	NA	NA	NA
	Double Difference	NA	NA	NA	NA
Steinman et al. (Unpublished Data)	Early	0.25	0.05	-0.002	0.08
	Late	0.17	-0.04	-0.10	0.02
	Whole Phase	0.24	0.00	-0.06	0.05
	Double Difference	0.05	0.08	0.10	0.05
	Early	NA	NA	NA	NA

Thompson et al. (63)	Late	NA	NA	NA	NA
	Whole Phase	NA	NA	NA	NA
	Double Difference	NA	NA	NA	NA
de Voogd et al. (66)	Early	NA	NA	NA	NA
	Late	NA	NA	NA	NA
	Whole Phase	NA	NA	NA	NA
	Double Difference	NA	NA	NA	NA
Wake et al. (39)	Early	0.001	0.12	0.09	0.12
	Late	-0.04	-0.06	0.05	-0.12
	Whole Phase	0.03	0.16	0.02	0.16
	Double Difference	-0.03	0.03	0.10	-0.02
Wake et al (69)	Early	-0.11	-0.07	-0.04	-0.07
	Late	0.30**	0.19	0.20	0.15
	Whole Phase	0.10	0.07	0.09	0.04
	Double Difference	-0.25*	-0.16	-0.15	-0.14

** Correlation significant at the 0.01 level (2-tailed)
*Correlation significant at the 0.05 level (2-tailed)

Supplementary Table S6.
Correlations between trait anxiety scores (STAI-T and STICSA) and difference scores (early, late, whole phase and double difference) for SCR during extinction

Study	Difference Score	STAI-T or STICSA
Goldfarb et al. (67)	Early	0.007
	Late	-0.121
	Whole Phase	-0.091
	Double Difference	0.082
Kanen et al. (68)	Early	-0.212
	Late	NA
	Whole Phase	NA
	Double Difference	NA
Lucas et al. (40)	Early	NA
	Late	NA
	Whole Phase	NA
	Double Difference	NA
Morriss et al. (36)	Early	-0.37
	Late	0.378
	Whole Phase	-0.029
	Double Difference	-0.477
Morriss et al. (37)	Early	-0.306
	Late	0.151
	Whole Phase	0.068
	Double Difference	-0.302
Morriss (35)	Early	0.236
	Late	-0.031
	Whole Phase	0.13
	Double Difference	0.208
	Early	0.227

Morriss & van Reekum (15)	Late	0.091
	Whole Phase	0.209
	Double Difference	0.136
Morriss & van Reekum (15)	Early	-0.011
	Late	0.082
	Whole Phase	0.053
	Double Difference	-0.065
Morriss & van Reekum (15)	Early	0.209
	Late	-0.088
	Whole Phase	0.118
	Double Difference	0.197
Morriss et al. (38)	Early	0.005
	Late	0.142
	Whole Phase	0.096
	Double Difference	-0.11
Sjouwerman et al. (64)	Early	-0.029
	Late	NA
	Whole Phase	NA
	Double Difference	NA
Sjouwerman et al. (65)	Early	-0.031
	Late	NA
	Whole Phase	NA
	Double Difference	NA
Sjouwerman & Lonsdorf (Unpublished Data)	Early	-0.106
	Late	NA
	Whole Phase	NA
	Double Difference	NA
Steinman et al. (Unpublished Data)	Early	0.218
	Late	0.111
	Whole Phase	0.189
	Double Difference	0.084
	Early	NA

Thompson et al. (63)	Late	NA
	Whole Phase	NA
	Double Difference	NA
De Voogd et al. (66)	Early	NA
	Late	NA
	Whole Phase	NA
	Double Difference	NA
Wake et al. (39)	Early	-0.005
	Late	-0.164
	Whole Phase	0.121
	Double Difference	-0.14
Wake et al (69)	Early	0.019
	Late	0.003
	Whole Phase	0.018
	Double Difference	0.012

** Correlation significant at the 0.01 level (2-tailed)
*Correlation significant at the 0.05 level (2-tailed)

To ensure that the null result for a relationship between SCR difference scores and self-reported variants of IU (IU-27, IU-12, I-IU and P-IU) during early extinction was not due to the inclusion of additional studies, meta-analyses for SCR difference scores during early extinction were reran with the 552 participants included in the meta-analyses of late extinction learning, the entire extinction phase and double difference scores. The null result remained for the relationship between SCR difference scores and of self-reported IU (IU-27, IU-12, I-IU, and P-IU) during early extinction learning (see, Supplementary Table 7).

Supplementary Table S7.
Pooled effect sizes and heterogeneity (percentage of variability in the effect size) for the IU subscales (IU-27, IU-12, I-IU, and P-IU) and SCR difference scores during early extinction learning, excluding participants not included in meta-analyses for SCR difference scores during late extinction learning, the entire extinction phase and double difference scores.

	g	(95% CI)	*k*	*N*	*p*	I^2
Early Ext						
IU-27	-0.032	(-0.21; 0.14)	12	504	.720	54.8%
IU-12	0.080	(-0.09; 0.25)	14	552	.352	49.9%
I-IU	0.037	(-0.13; 0.206)	14	552	.665	44.6%
P-IU	0.089	(-0.08; 0.26)	14	552	.296	48.3.%

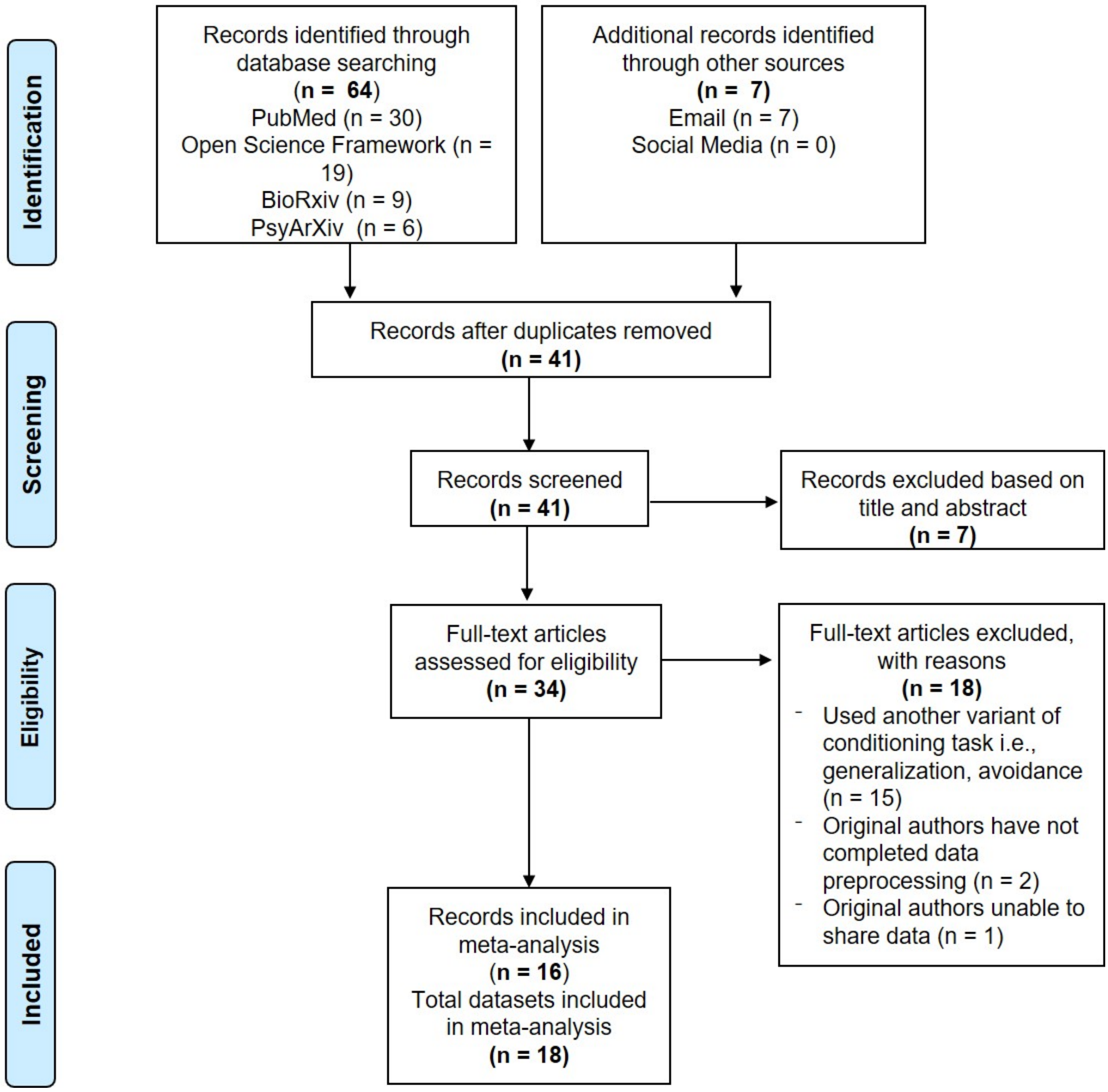

Figure S1. Flowchart
